# Notes on Preparations for the Sick

**Published:** 1906-12

**Authors:** 


### Notes on Preparations for the Sick.

**Peptones of Beef, Milk and Wheat** (Allenbury's).—Allen and Hanburys, London.—This combination of the albuminoids of beef, milk and wheat forms a nourishing predigested food which cannot fail to be of use in cases of debility arising from a multitude of causes. It is considered to be an ideal restorative for convalescents, invalids and the aged. It should be given in quantities of one to two tablespoonfuls as often as may be required. It is a clear but dark-coloured, palatable fluid, which gives no precipitate with nitric acid, and only a slight one with picric acid. Being predigested with pancreatic ferments, it should afford an easily assimilated nourishment in cases of gastric and digestive insufficiency from whatever cause.

---

**The V.P. Grape Juice Cordial.**—The Vine Products Co., 74 Great Tower Street, London.—This is a non-alcoholic drink prepared from pure grape juice, and containing no injurious preservative. The flavour is excellent, and diluted with aerated water it makes a very palatable and refreshing drink. The sweetness of the fluid is entirely due to grape sugar; no sugar is added. It makes an excellent drink both for summer and for winter use, either cold with ærated water, or diluted with about five times its bulk of hot water. It should be much appreciated by those who do not take the products of the grape after the ordinary fermentation. It will keep well, inasmuch as the high percentage of grape sugar (50 per cent.) prevents fermentation, and the other chemical constituents of this condensed grape extract should have a diuretic effect, and be of service in counteracting dyspepsia and constipation.

---

**Leukion** (Evans): Ung. Iodi. Decolorata.—Steele and Marsh, Bath.—This new preparation of iodine for external uses is certified by Mr. Gatehouse to contain free iodine to the extent of five per cent. Its especial virtue is due to the fact that when rubbed into the skin the black colour disappears; this fact must make it acceptable to the patient for many purposes where the staining properties of the ordinary iodine preparations are a great disadvantage. It is a black, soft and odourless ointment, which should be readily absorbed through the skin and without giving rise to any irritation, discoloration or desquamation. Its numerous uses locally are as extensive as those of iodine itself, and the only instruction for its use is that it should be rubbed into the part needing it until the colour disappears.

---

**Tabloids.**—Burroughs, Wellcome & Co., London.—**Tabloid Xaxa and Dover Powder.**—Each contains: Xaxa, gr. $2\frac{1}{2}$; Dover Powder, gr. $2\frac{1}{2}$. Xaxa is acetyl-salicylic acid, is widely recognised as a valuable anti-rheumatic, antipyretic, antiseptic and analgesic. Its range of usefulness is extended by combination with Dover's Powder. This compound is indicated in influenza, catarrh, and in various febrile and infective conditions. In such affections it reduces temperature and relieves pain. One to four tabloids may be swallowed with a little water as may be necessary.

**Tabloid Trinitrin.**—Tabloid Trinitrin should be highly appreciated by the medical profession as a reliable means of administering exact doses of the drug. Reliability of medicament is of the first importance in aortic disease and in attacks of angina pectoris, when prompt action is essential. The portability of the tabloid enables the patient always to have the remedy in his pocket, ready for instant use in case of an attack. Trinitrin is one of the many important medicinal agents in the introduction of which Burroughs, Wellcome & Co. were pioneers.

LIST OF PREPARATIONS.

| | |
|---|---|
| Tabloid Trinitrin ... ... ... | gr. $\frac{1}{200}$ |
| " " ... ... ... | " $\frac{1}{100}$ |
| " " ... ... ... | " $\frac{1}{50}$ |
| Tabloid Trinitrin Compound: | |
| ℞ Trinitrini ... ... ... | gr. $\frac{1}{100}$ |
| Capsicini . ... ... | " $\frac{1}{200}$ |
| Menthol ... ... ... | " $\frac{1}{100}$ |
| Tabloid Hypodermic Trinitrin ... | gr. $\frac{1}{250}$ |
| " " " ... | " $\frac{1}{100}$ |

**Soloids, for ophthalmic use.**—When it is desired to obtain a solution for ophthalmic purposes, the Soloid products will be found valuable. They permit of the ready preparation of small amounts of appropriate strengths, *e.g.* one Soloid Atropine Sulphate gr. 0.545, dissolved in one drachm of distilled water, yields a one per cent. solution. The use of the Soloid products ensures active medicament and fresh reliable solution, prevents waste, and obviates the use of preservatives (which are specially undesirable in eye lotions).

| | |
|---|---|
| Soloid Atropine Sulphate ... ... | gr. 0.545 |
| " " " ... ... | " 0.272 |
| " Cocaine Hydrochlor. ... | " 1.09 |
| " Homatropine Hydrobrom. ... | " 0.545 |
| " Copper Sulphate ... ... | " 1 |

Tabloid ophthalmic products readily dissolve when placed on the conjunctiva and quickly exert their effect. They are easily applied and widely employed. The following new strengths are now issued:—

| | |
|---|---|
| Tabloid Ophthalmic Physostig. Salicyl | gr. $\frac{1}{2000}$ |
| Tabloid Ophthalmic Dionine ... | gm. .005 |

